# Reduced-cost *Chlamydia trachomatis*-specific multiplex real-time PCR diagnostic assay evaluated for ocular swabs and use by trachoma research programmes

**DOI:** 10.1016/j.mimet.2017.04.010

**Published:** 2017-08

**Authors:** Robert Butcher, Jo Houghton, Tamsyn Derrick, Athumani Ramadhani, Beatriz Herrera, Anna R. Last, Patrick A. Massae, Matthew J. Burton, Martin J. Holland, Chrissy h. Roberts

**Affiliations:** a Clinical Research Department, Faculty of Infectious and Tropical Diseases, London School of Hygiene & Tropical Medicine, London WC1E 7HT, United Kingdom; b Kilimanjaro Christian Medical Centre, Moshi, Tanzania

**Keywords:** Chlamydia trachomatis, Diagnosis, Quantitative PCR, Trachoma

## Abstract

**Introduction:**

Trachoma, caused by the intracellular bacterium *Chlamydia trachomatis* (*Ct*), is the leading infectious cause of preventable blindness. Many commercial platforms are available that provide highly sensitive and specific detection of *Ct* DNA. However, the majority of these commercial platforms are inaccessible for population-level surveys in resource-limited settings typical to trachoma control programmes. We developed two low-cost quantitative PCR (qPCR) tests for *Ct* using readily available reagents on standard real-time thermocyclers.

**Methods:**

Each multiplex qPCR test targets one genomic and one plasmid *Ct* target in addition to an endogenous positive control for *Homo sapiens* DNA. The quantitative performance of the qPCR assays in clinical samples was determined by comparison to a previously evaluated droplet digital PCR (ddPCR) test. The diagnostic performance of the qPCR assays were evaluated against a commercial assay (*artus C*. *trachomatis* Plus RG PCR, Qiagen) using molecular diagnostics quality control standards and clinical samples. We examined the yield of *Ct* DNA prepared from five different DNA extraction kits and a cold chain-free dry-sample preservation method using swabs spiked with fixed concentrations of human and *Ct* DNA.

**Results:**

The qPCR assay was highly reproducible (*Ct* plasmid and genomic targets mean total coefficients of variance 41.5% and 48.3%, respectively). The assay detected 8/8 core specimens upon testing of a quality control panel and performed well in comparison to commercially marketed comparator test (sensitivity and specificity > 90%). Optimal extraction and sample preservation methods for research applications were identified.

**Conclusion:**

We describe a pipeline from collection to diagnosis providing the most efficient sample preservation and extraction with significant per test cost savings over a commercial qPCR diagnostic assay. The assay and its evaluation should allow control programs wishing to conduct independent research within the context of trachoma control, access to an affordable test with defined performance characteristics.

## Highlights

• Current WHO guidelines for trachoma control do not recommend tests for infection.
• A *Chlamydia trachomatis* diagnostic more suited to low-income economies was developed.
• A complete sample-to-result pipeline is evaluated.
• The assay offers high throughput and comparable performance to commercial diagnostics.
• The assay was significantly cheaper than commercial alternatives.

## Graphical abstract

**Figure:**
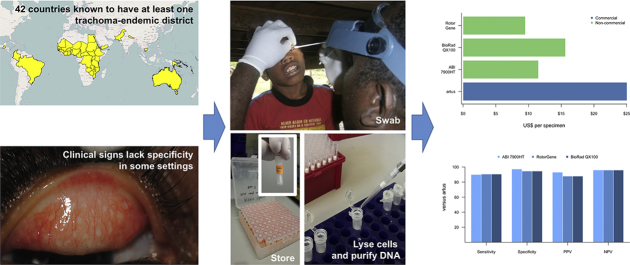

## 1 Introduction

*Chlamydia trachomatis* (*Ct*) is the cause of trachoma, which is the leading cause of infection-related blindness worldwide (Taylor, 2008, Bourne et al., 2013). *Ct* is also the most commonly diagnosed bacterial sexually transmitted infection (World Health Organization, 2012). Diagnosis of trachoma is made by the observation of a clinical sign which is the appearance of lymphoid follicles and inflammation on the tarsal conjunctiva (Solomon et al., 2004a). This clinical sign is not highly specific (Burton et al., 2003) and in low-prevalence or post-treatment settings can correlate poorly with for ocular *Ct* infection (Baral et al., 1999, Burton et al., 2010, Ramadhani et al., 2016). Control programs use azithromycin mass drug administration (MDA) in trachoma endemic communities as part of an overall strategy to control transmission, but the drop in prevalence of infection results in a decrease in the positive predictive value of clinical signs of disease (Ramadhani et al., 2016).

Determination of infection load data offers additional benefits to a qualitative diagnostic assay because load of infection is associated with disease severity (Burton et al., 2003). Reference-free methods for quantitation of nucleic acids using digital droplet PCR (ddPCR) technology have also been evaluated (Roberts et al., 2013); these have been useful in demonstrating that infection load may be involved in transmission (Last et al., 2017). In populations that have been treated *en masse* with azithromycin, the loads of individual infections are usually low (Solomon et al., 2004b). Identifying sub-populations in which there are infections with higher than average loads can identify communities and subgroup ‘hotspots’ that are reservoirs of infection in otherwise trachoma-free areas (Alexander et al., 2005). Conversely, infections in low-prevalence or post-treatment settings may not be of high enough load to sustain transmission and the community or burden of infection may decline; this is referred to as the Allee effect (Burton et al., 2010, Solomon et al., 2008). A quantitative diagnostic test may therefore assist in programmatic decisions such as when to continue, cease or target azithromycin MDA (Yohannan et al., 2013).

Nucleic acid amplification tests (NAATs) have become the gold standard for *Ct*-specific tests of infection, due to their superior sensitivity and throughput when compared to culture and antigen detection techniques (Papp et al., 2014). There are many accredited commercial assays for the diagnosis of sexually transmitted *Ct* infections but very few are evaluated for testing with ocular swabs. Diagnostic tests with quantitative capabilities, such as the Abbott RealTi*m*e CT/NG *m*2000 (Cheng et al., 2011) platform is widely distributed in many low- and middle-income countries yet per-specimen testing costs remain beyond many trachoma control and research programs.

Nucleic acid amplification tests for *Ct* are not currently required by the international guidelines for implementation or cessation of the “SAFE” (**S**urgery for the correction of in-turned eyelashes, **A**ntibiotics to treat infection, promotion of **F**acial hygiene and **E**nvironmental improvement to reduce transmission) strategy for trachoma control (World Health Organization, 2010). Diagnosis of current *Ct* infection can be a valuable component of the monitoring and evaluation of trachoma control programs (Solomon et al., 2004c). Where NAATs have been used, both commercial (Harding-Esch et al., 2013) and non-commercial (Butcher et al., 2016, Macleod et al., 2016) tests have yielded important results that have shaped the scientific agenda for control program evaluation, the research activities of government/non-government organisations and the policy of funding bodies. The cost efficacy of using a commercial NAAT to guide MDA cessation has been evaluated (Harding-Esch et al., 2015) and it was found that a low-cost commercial NAAT can be cost effective for the control program as test costs were offset by savings from the distribution of further unnecessary annual treatment rounds of MDA. In addition to the cost benefit, avoiding unnecessary rounds of MDA would reduce community antibiotic exposure and risk of emergence of antibiotic resistance.

There are many situations in which an open-platform test with evaluated performance characteristics may enable valuable data to be gathered and we therefore designed and evaluated the performance of a NAAT for detection and enumeration of ocular *Ct* infections. The test was required to be high throughput, low cost, quantitative and comparable in performance to a commercial alternative. Capacity to multiplex targets was also important to enable concurrent testing of specimen collection and extraction. qPCR was therefore selected over other technologies suitable for low-resource settings (such as end-point PCR or loop-mediated amplification (LAMP) (Choopara et al., 2017)).

## 2 Methods

### 2.1 Study ethics

Samples were collected from trachoma-endemic communities in Tanzania and Guinea-Bissau as detailed below. Ethical approval for the collection of these samples was obtained from the following ethics committees; Comitê Nacional de Ética e Saúde (Guinea Bissau), London School of Hygiene & Tropical Medicine (LSHTM; UK), Kilimanjaro Christian Medical Centre and the National Institute for Medical Research (Tanzania). The support of local leaders in every community was ascertained before sample collection began. All participants were required to provide written, informed consent prior to study enrollment and parents or guardians provided consent for children.

### 2.2 Oligonucleotides

Primer and hydrolysis probe sequences used in this study are shown in Table 1. Primers targeting highly conserved species-specific regions of plasmid open reading frame 2 (pORF2) and outer membrane protein complex B (*omcB*) of *Ct* were previously described by Pickett and colleagues (Pickett et al., 2005). pORF2 and *omcB* probe sequences were designed using Primer Express v3 (Life technologies, Paisley, UK). Oligonucleotides for use on Applied Biosystems (ABI) real-time thermocyclers were synthesized by Life Technologies. Oligonucleotides for use on the Corbett Rotor-Gene (also known as Qiagen Rotor-Q) were synthesized by Sigma (Sigma-Aldrich, UK). Endogenous control primers and probes specific to the *Homo sapiens* RNase P/MRP 30-kDa subunit (*RPP30*) gene were previously described by Luo and colleagues (Luo et al., 2005). There is no variation in primer or probe binding sites in published *Ct* genome and plasmid sequences (NCBI Blastn search January 2017). *omcB* and pORF2 targets are present in a single copy per chlamydial genome and plasmid, respectively. Studies have estimated the median plasmid copy number in clinical specimens to be approximately five copies per genome (range: 1–18) (Last et al., 2013).

**Table 1:** Olignucleotides used in this study.

Target	Oligo	Sequence (5′-3′)	Amplicon size (bp)
*C*. *trachomatis omcB*	Primer (F)^a^	GAC ACC AAA GCG AAA GAC AAC AC	106
	Primer (R)^a^	ACT CAT GAA CCG GAG CAA CCT	
	ABI – Probe^b^	[FAM]-CCA CAG CAA AGA GAC TCC CGT AGA CCG-[QSY]	
	Rotor-Gene - Probe^b^	[FAM]-CCA CAG CAA AGA GAC TCC CGT AGA CCG-[BHQ]	
*C*. *trachomatis* pORF2	Primer (F)^a^	CAG CTT GTA GTC CTG CTT GAG AGA	109
	Primer (R)^a^	CAA GAG TAC ATC GGT CAA CGA AGA	
	ABI Probe^b^	[NED]-CGG GCG ATT TGC CTT-[MGBNFQ]	
	Rotor-Gene - Probe^b^	[JOE]-CCC CAC CAT TTT TCC GGA GCG A-[BHQ1]	
*H*. *sapiens RPP30*	Primer (F)^a^	AGA TTT GGA CCT GCG AGC G	65
	Primer (R)^a^	GAG CGG CTG TCT CCA CAA GT	
	ABI – Probe^b^	[VIC]-TTC TGA CCT GAA GGC TCT GCG CG-[QSY]	
	Rotor-Gene – Probe^b^	[Cy5]-TTC TGA CCT GAA GGC TCT GCG CG-[BHQ2]	

ABI: Applied Biosystems; Bp: base pairs; F: forward; *omcB*: outer membrane protein complex B; pORF2: plasmid open reading frame 2; R: reverse; RPP30: RNase P/MRP 30-kDa subunit.

a Primers purified by desalting.

b Probes purified by high-performance liquid chromatography.

### 2.3 qPCR

For ABI thermal cyclers, each 10-μL qPCR contained final concentrations of 1 × TaqMan Universal Mastermix II, with Uracil-DNA N-glycosylase (UNG, a common method to minimize PCR cross-contamination by enzymatic degradation of previous PCR products with incorporated dUTP; Life technologies, Paisley, UK), each oligonucleotide at 0.3 μM and 2 μL template DNA in aqueous solution. In the UK, the assay was performed on an ABI 7900HT Fast Real Time PCR machine (Life Technologies, Paisley, UK). In Tanzania, the assay was performed on an Applied Biosystems ViiA 7 Real Time PCR machine (Life Technologies, Paisley, UK). Both instruments utilized a 384-well format.

For Rotor-Gene thermal cyclers samples were run in a 72-well rotor format on a Corbett Rotor-Gene 3000. Each 20-μL qPCR contained 1 × qPCRBIO Probe Mix No-Rox (PCR Biosystems, London, UK), each oligonucleotide at 0.3 μM and 8 μL template DNA in aqueous solution.

No-template controls and serial dilutions of known-concentration PCR product were included on each run on all systems. Thermal cycling conditions for all systems were 50 °C for 2 min, 95 °C for 10 min, followed by 45 cycles of 95 °C for 15 s and 60 °C for 60 s. Samples with quantitation cycle (C_q_) values < 15 cycles were diluted and retested.

### 2.4 Calibration curve

Calibration standards were prepared using DNA extracted from cultured Human Epithelial type-2 (HEp-2) cells, which had been infected with *Ct* strain A2497. The Novogen KOD PCR kit (Novogen, Sydney, Australia) was used to amplify the pORF2, *omcB* and *RPP30* targets using the primers described in Table 1. PCR products were purified and extracted using the Promega gel clean-up kit and Wizard SV PCR spin columns (Promega, Madison, USA) according to manufacturer's guidelines. PCR products were then diluted 1:10^7^ in 1 mM Tris-Cl 0.1 mM EDTA (0.1 × TE) buffer on a background of 2 ng/μL herring sperm DNA (Sigma Aldrich, St Louis, USA). These standards were ten-fold serially diluted through ten steps to create a calibration curve. A ddPCR assay (Last et al., 2013, Roberts et al., 2013) was used to estimate the number of chlamydial and human targets in each standard. The limit of detection was defined as the lowest analyte concentration at which all ten repeat measurements of a specific dilution returned a positive result.

### 2.5 Analysis of clinical samples

To assess the performance of the qPCR test in clinical specimens, we compared the results of testing by *artus C. trachomatis* Plus RG PCR, qPCR and ddPCR. This analysis used 99 randomly selected samples from a collection of clinical ocular-swab derived DNA specimens that originated from a 2014 study of children aged 1–9 years selected from trachoma-endemic communities on the Bijagos Islands, Guinea-Bissau. Sample collection protocols and *Ct* ddPCR results were described previously (Derrick et al., 2016). In Tanzania we also tested 523 samples with qPCR referenced against ddPCR. These clinical samples were from a single cross-sectional time point of a 4-year longitudinal study of a cohort of children aged 5–10 years of age. In each study, samples were collected prior to community treatment with azithromycin.

The *artus C. trachomatis* Plus RG PCR Kit (96) CE (Qiagen, Manchester, UK; catalogue number: 4559265) was used to test the samples from the Guinea-Bissau cohort. Testing was performed as per manufacturer's instructions using the kit internal control to monitor possible PCR inhibition by adding directly to the reaction mixture.

### 2.6 Quality control molecular diagnostics panel performance

Using the assays described above, we used an external quality control molecular diagnostics panel of samples, (Quality Control in Molecular Diagnostics (QCMD) programme (www.qcmd.org) (Staines et al., 2009)). The panel consisted of positive and negative samples which participants were expected to detect (termed ‘core’ samples) and low-load samples termed ‘educational’ that contained < 1 genome equivalent per microlitre of sample. In ‘educational’ specimens, the load is so low that only extremely sensitive tests (i.e. those using target enrichment or transcription-assisted amplification) would be expected to routinely identify these as positive. The lyophilized samples were rehydrated according to QCMD protocol, in 4 mL of sterile molecular biology-grade water, of which DNA was extracted from 1 mL and eluted into 100 μL. Following DNA extraction, the assays were performed as described.

### 2.7 Testing costs of extracted DNA from clinical swabs by *artus C. trachomatis* Plus RG PCR, qPCR and ddPCR

The laboratory cost of processing the samples by each assay was calculated for both personnel time and consumables. Costs of consumables were taken from current UK list prices at the time of publication and were expressed in US Dollars (US$). Personnel costs were based on a salary for a junior laboratory technician at Kilimanjaro Christian Medical Centre in Moshi, Tanzania. Per-sample costs were calculated based on the time taken to complete one run on each analysis platform and then expressed as a per sample cost including overheads. Equipment procurement costs were not included in the analysis, as thermocycler availability and use will vary between country and laboratory. However, prices for the thermocyclers used in this manuscript are stated in the footnote for Table 6.

### 2.8 Preparation of ‘spiked’ swabs for use in storage assessment experiments

A suspension of cultured HEp-2 cells and serovar A *Ct* EBs in phosphate-buffered saline (PBS) was inoculated onto swabs. HEp-2 cells were spiked into PBS at approximately 400,000 per 1 mL of PBS. Elementary bodies (EBs) were spiked into the same suspension at a dilution of 2 μL of EBs per 1 mL of PBS to achieve a high concentration of *Ct* targets per PCR reaction. The suspension was homogenized and 50 μL was aliquoted onto polyester-coated swabs (Puritan Medical Products, Guilford, USA). Swabs were allocated to storage in one of three conditions: dry storage in polystyrene tubes at room temperature (uncontrolled, typically 22–25 °C), dry storage in paper envelopes at room temperature inside a domestic vacuum-sealed container with silica desiccant, or dry storage in polystyrene tubes at − 20 °C. Four swabs were prepared and processed per time point per storage condition. Swabs were removed from storage at 7, 30, 90 and 180 days and DNA was extracted using the swab protocol of the QIAamp DNA mini kit (Qiagen, Manchester, UK) according to manufacturer's instructions. Swabs were tested using ABI qPCR.

### 2.9 Comparison of DNA extraction kits and recovery of Chlamydia trachomatis nucleic acids

Peripheral blood mononuclear cells (PBMCs) were extracted from the blood of a healthy volunteer. PBMCs were suspended in PBS and aliquots spiked with high, medium and low loads of *Ct* A2497 EBs. One aliquot did not have any EBs added to act as negative control. A 50-μL aliquot of suspension was pipetted directly onto swabs. A total of 25 swabs were prepared at each concentration level and refrigerated overnight. Five swabs were selected at random from each concentration group. Each swab was rehydrated in 400 μL of PBS. They were then vortexed at full speed for 2 min and the swab was removed and discarded, expressing any excess liquid on the side of the tube. DNA was then prepared following the manufacturer's recommendations for each respective kit. The elution volume was standardised to 100 μL. Five extraction kits were compared: MTB Isolation (Elisabeth Pharmacon), Blood and Serum DNA Isolation Kit (BioChain), Cador Pathogen (Qiagen), QIAamp Mini DNA Extraction (Qiagen) and Power Soil DNA Isolation Kit (MoBio), which includes a mechanical lysis step (specimen lysed in PowerBead tube at 6 m/s for 40 s). Four 1 μL aliquots of eluate were tested per swab, resulting in 20 test wells per condition. C_q_ values from high-, medium- and low-load sample eluates were collated into a single dataset for each extraction kit. QIAmp DNA mini kit was used as our standard reference as it is used widely in many studies, and our group has used this kit extensively in trachoma studies (Roberts et al., 2013, Solomon et al., 2003, Burton et al., 2011).

### 2.10 Data analysis

Data were reported in accordance with Minimum Information for Publication of Quantitative Real-Time PCR Experiments (MIQE) guidelines (Bustin et al., 2009). Clinical specimens and PCR product dilutions were classified as positive for *Ct* if the test detected amplification of the plasmid target in any well within 40 cycles for the ABI assay, or 35 cycles for the Rotor-Gene assay. The load of infection was determined by extrapolation from an eight-step, ten-fold dilution of PCR product standards of known concentration; these were tested in triplicate on each plate.

SDS 2.4 software (Life Technologies, Paisley, UK) was used for data analysis. Baseline fluorescence intensity values were determined by analysis of mean fluorescence between cycles 3 and 15 on both platforms. The C_q_ boundary line was set at 0.2 for all three fluorescence channels (FAM/VIC/NED) on the ABI instrument, and at 0.1 on the Rotor-Gene instrument. C_q_ data were exported from respective data collection software and further analysed using R version 3.2.2 (R Core Team, 2014). Linear regression was used to determine whether the C_q_ decreased significantly with time under different storage conditions. The gradients of linear models were examined to determine whether a significant downward trend was identified. To determine if there were significant differences between *Ct* DNA recovery from extraction kits, homogeneity of variance within the total dataset was assessed using Fligners test. One-way Analysis of Variance (ANOVA) with Tukey's Honest Significant Difference (HSD) post-hoc test was used to determine which of the observed differences were significant.

## 3 Results

### 3.1 Assay performance

The assay characteristics derived from repeat testing of a standard curve are presented in Table 2. The experimentally determined dynamic range of the assay was between 10^0^ and 10^6^ copies of *omcB* and/or pORF2 per reaction. The coefficients of determination for all three targets in both assays was > 0.99. Amplification of all targets was highly (> 95%) efficient. *omcB*, pORF2 and *RPP30* were reproducibly detected at concentrations of 0.9–8.3 copies per test, but not below. No-template controls tested negative on every run.

**Table 2:** Assay characteristics derived from repeat-tested standard curve.

Assay	Target	CoV	Gradient	CoD	Efficiency (%)	LoD	C_q_ range at LoD
ABI 7900HT	pORF2	41.5	− 3.4	0.990	96.7	8.3	34.9–37.0
	*omcB*	48.3	− 3.3	0.998	100.1	4.5	37.4–39.7
Rotor-Gene 3000	pORF2	20.3	− 3.3	0.999	100.2	0.9	30.0–31.1
	*omcB*	35.3	− 3.3	0.999	100.2	1.4	31.5–32.6

C_q_: quantitation cycle; CoD: coefficient of determination; CoV: coefficient of variance; LoD: limit of detection in copies/reaction; *omcB*: outer membrane protein complex B; pORF2: plasmid open reading frame 2.

The mean coefficient of variance around all data points across the whole dynamic range was 48.3% for the *omcB* target, and 41.5% for the pORF2 target, approximately equivalent to 0.6 and 0.5 PCR cycles, respectively. For the Rotor-Gene assay, the coefficient of variance was 35.3% for *omcB* and 20.3% for pORF2, equivalent to approximately 0.4 and 0.3 cycles, respectively. The largest contributor to assay variance on both platforms was between-run variation. The coefficient of variance generally increased at lower concentrations, possibly reflecting the increased chance of sampling handling error where analytes are rare. Interestingly, when Cq values were compared between ABI 7900HT and ABI Viia7 machines, the assay parameters were mostly similar with the exception that absolute *omcB* C_q_ values were consistently between 0.5 and 1.5 cycles higher when tested in Tanzania than when tested in the UK.

### 3.2 Quality control panel performance

All three assays (*artus*, ABI and Rotor-Gene) performed well when used to test external quality control molecular diagnostics samples, correctly diagnosing 8/8 (100%) of ‘core’ samples. Two low-load ‘educational’ samples, which were below the measured limit of reproducible detection for the assay were not classified as positive. The results are shown in Table 3.

**Table 3:** External quality assessment panel performance of qPCR assays.

Sample ID	Sample	Sample type	QCMD concentration (copies/mL)	*artus* qualitative result	ABI-qPCR qualitative result	Rotor-Gene qualitative result
CTA13-01	*Ct*^+^ urine	Core	Positive (250)	Positive	Positive	Positive
CTA13-02	*Ct*^+^ urine	Educational	Positive 63	Not determined	Negative	Positive
CTA13-03	nv*Ct*^+^ urine	Core	Positive 10,000	Positive	Positive	Positive
CTA13-04	*Ct*^+^ urine	Core	Positive 1000	Positive	Positive	Positive
CTA13-05	*Ct*^−^ urine	Core	Negative 0	Negative	Negative	Negative
CTA13-06	nv*Ct*^+^ urine	Core	Positive 10,000	Positive	Positive	Positive
CTA13-07	*Ct*^+^ swab	Educational	Positive 13	Positive	Negative	Positive
CTA13-08	*Ct*^*−*^ swab	Core	Negative 0	Negative	Negative	Negative
CTA13-09	*Ct*^+^ swab	Core	Positive 250	Positive	Positive	Positive
CTA13-10	*Ct*^+^ swab	Core	Positive 63	Positive	Positive	Positive
		Core performance		8/8	8/8	8/8
		Educational performance		2/2	0/2	2/2

*Ct*: *Chlamydia trachomatis*.

### 3.3 Clinical specimens

**Fig. 1:**
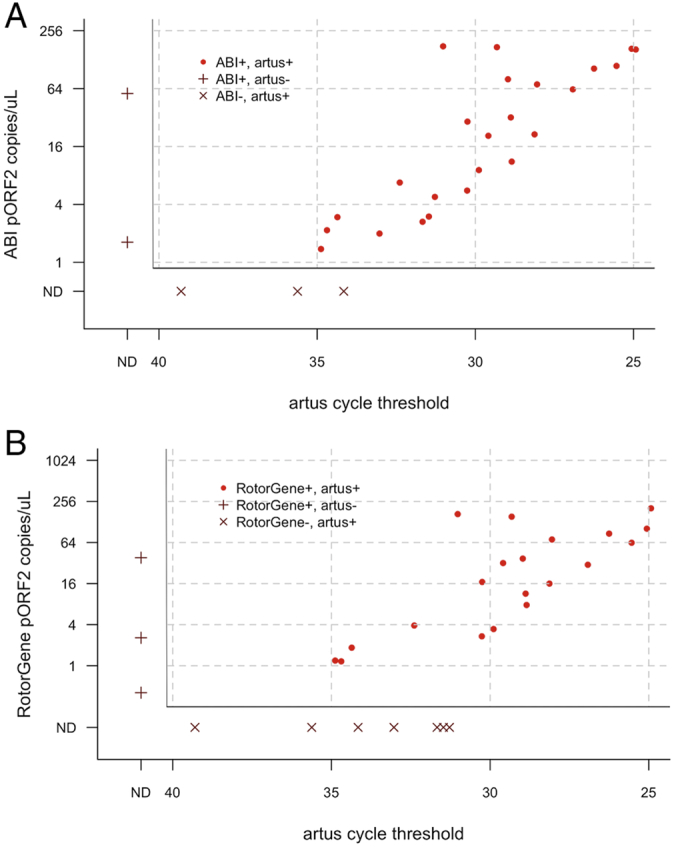
Comparison of plasmid load estimate from qPCR compared to *artus* cycle threshold. (A). ABI 7900HT. (B). Rotor-Gene 3000. The main plots show concordant results (ABI *n* = 24, Rotor-Gene *n* = 20), side panels show discordant results. ND: Not detected.

**Fig. 2:**
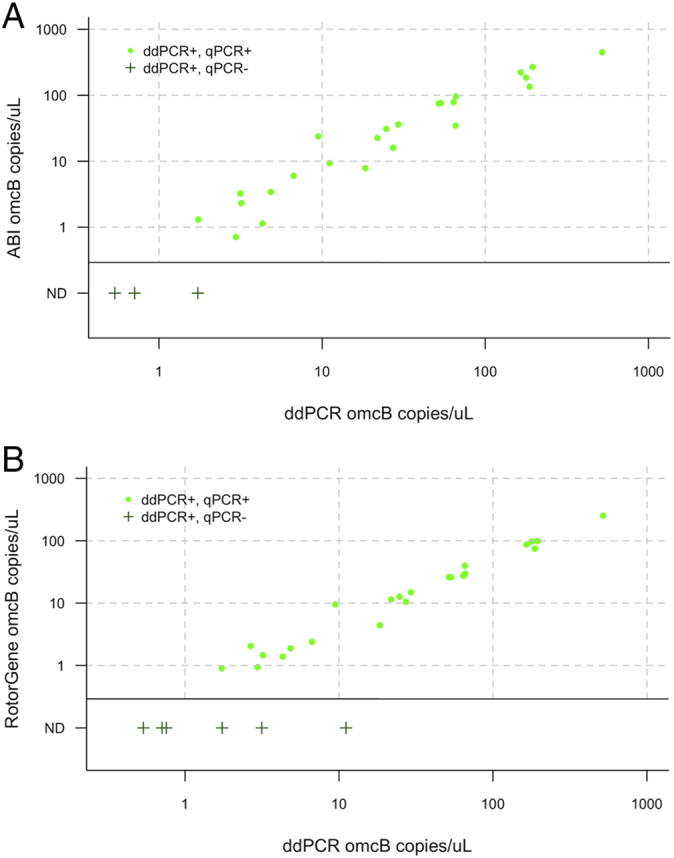
Agreement between load estimates from ddPCR and qPCR. (A) ABI 7900HT. (B) Rotor-Gene 3000. Main panels show concordant results, side bars show discrepant results. ND: Not detected.

According to the validated commercial kit (*artus*), 28/99 Guinea Bissau specimens were positive for *Ct*. The mean C_q_ of those positive specimens was 30.3. On the ABI platform 26/99 of the Guinea Bissau samples were positive. Of the same sample set, 23/99 samples tested positive by Rotor-Gene. Of the *artus*-positive results, only one was negative by all other methods. The load estimates from samples where both targets were detected is shown in Fig. 1. *omcB* was not detected in two of the ABI positive specimens, which had a plasmid load of 2 and 3 copies/μL, respectively. The median *artus* C_q_ of *artus*+ qPCR- (false negative) specimens was 34.9 and 32.4 cycles for the ABI and Rotor-Gene assays, respectively (Fig. 1). The median load of *artus*-qPCR+ specimens was 29 and 3 copies/μL for the ABI and Rotor-Gene assays, respectively. For *omcB*, the median load of the ddPCR+ qPCR- samples was 1.2 and 0.7 copies/μL for the ABI and Rotor-Gene platforms, respectively (Fig. 2). There were no ddPCR- qPCR+ samples.

In Tanzania we tested a further 523 clinical samples by ABI qPCR that had also been tested by ddPCR at LSHTM. The overall prevalence of infection by ddPCR was 12.4% (65/523). By qPCR there were 78/523 positive samples leading to a sensitivity and specificity of 100% (95% CI 94.5–100) and 97.2% (95% CI 95.2–98.5). A summary of sensitivity and specificity data are shown in Table 4.

**Table 4:** Diagnostic comparison of noncommercial qPCR assays to commercially marketed comparator.

	*artus C. trachomatis* Plus RG PCR versus⁎		
	qPCR (ABI 7900HT)	qPCR (Rotor-Gene 3000)	ddPCR (Bio-Rad QX100)
Sensitivity	90 (73.5–97.9)	90.6 (75–98)	90.6 (75–98)
Specificity	97.3 (96.0–99.7)	94.6 (86.7–98.5)	94.6 (86.7–98.5)
PPV	93.1 (77.4–98.6)	87.9 (73.5–95)	87.9 (73.5–95)
NPV	96 (89.2–98.6)	95.9 (88.8–98.6)	95.9 (88.8–98.6)
Cohens Kappa	0.82	0.78	0.83

NPV: Negative predictive value; PPV: positive predictive value.

⁎ 95% confidence intervals shown in brackets.

### 3.4 Comparative efficiency of sample extraction and yield

**Fig. 3:**
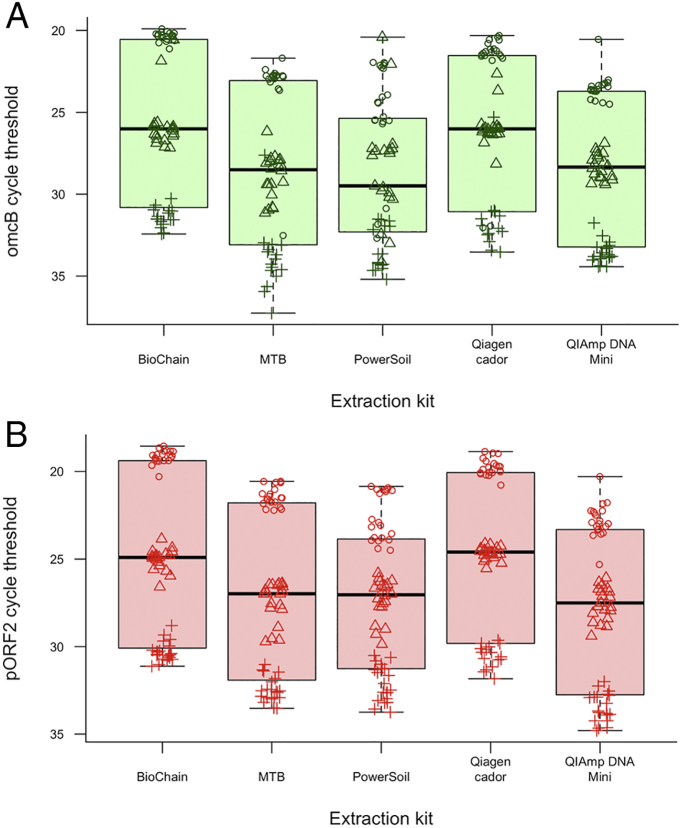
Recovery of (A) *omcB* and (B) pORF2 from *Ct*-spiked swabs by five different extraction kits. Boxes represent median, inter-quartile range and range of all swabs for each treatment. Circles represent swabs spiked with high-load elementary bodies, triangles represent medium-load spiking, and crosses represent low-load spiking. There is variation in the number of targets recovered by different extraction kits. Biochain and Qiagen cador kits appear to yield more *Ct* DNA than comparators.

DNA prepared using the PowerSoil DNA kit yielded the most variable estimates of *Ct* burden overall (Fig. 3). Qiagen Cador and Biochain kits recovered the highest amounts of *Ct* DNA measured by the quantity of *omcB* (*p* = 0.001 and *p* = 0.0004, respectively) and pORF2 (*p* = 0.0004 and *p* = 0.002, respectively) load compared to QIAmp DNA mini extraction. Using one-way ANOVA was considered appropriate as there was no significant heterogeneity in the variance between comparator groups (Fligner's test (*omcB p* = 0.31 and pORF2 *p* = 0.66)). There were significant differences within the model for both targets (*omcB p* = 0.00005 and pORF2 *p* = 0.00003). Pair-wise analyses using Tukey's HSD *post*-*hoc* test, found that both Qiagen Cador and Biochain kits had significantly higher yield when compared to MTB, QIAmp DNA mini and PowerSoil DNA kits. The results of the pair-wise comparisons were consistent for both *omcB* and pORF2 targets.

### 3.5 Sample preservation and storage

**Fig. 4:**
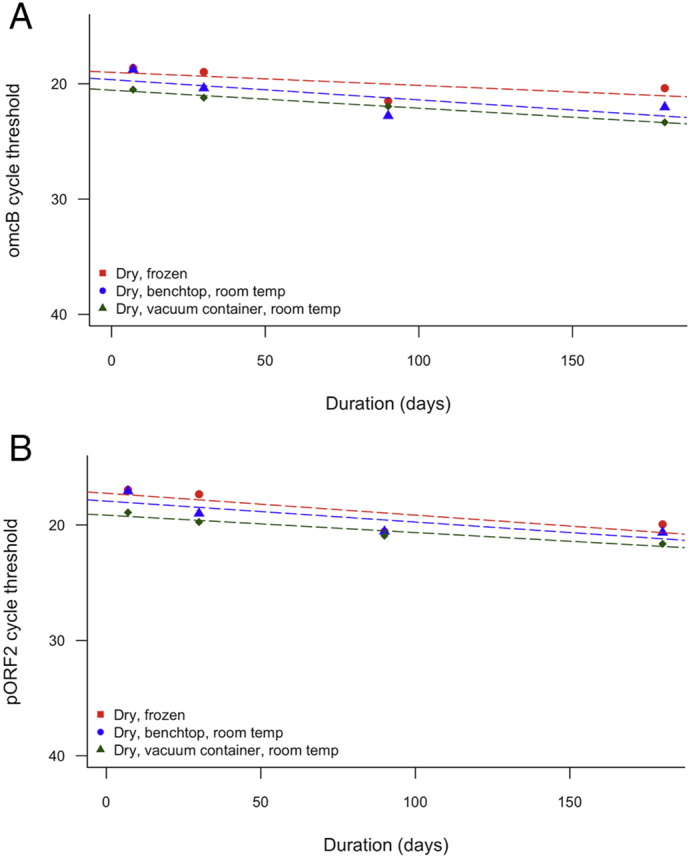
Change in recovered load of (A) *Ct* plasmid and (B) genomic targets following long-term storage frozen and at room temperature. Points represent mean of four swabs per time point per condition. Dashed lines represent linear regression model between load and time.

*Ct* and human DNA was readily detectable in all samples at all time points, with no diagnostic failures by 6 months storage at room temperature (Fig. 4). All three treatments showed a significant increase in C_q_ required to detect *Ct* over 6 months according to linear regression models, indicating a decrease in target abundance (Table 5). Based on these models the estimated rate of reduction in detectable load was 0.01–0.02% of the 7-day specimen C_q_ per day. After 6 months, the mean C_q_ for detection of plasmid had increased by 18% for the frozen swabs, and by 21% and 14%, respectively, for the desktop and vacuum contained room temperature swabs. The C_q_ for *omcB* target detection had increased by 9% for the frozen swabs, and by 17% and 14%, respectively, for the desktop and vacuum contained room temperature swabs.

**Table 5:** Coefficients from linear regression models examining the relationship between cycle threshold and time in days.

Treatment	*Ct* pORF2		*Ct omcB*	
	Gradient	*p*-value	Gradient	*p*-Value
Dry, frozen	0.019	< 0.0001	0.011	< 0.0001
Dry, desktop, room temperature	0.018	< 0.0001	0.018	< 0.0001
Dry, vacuum box, room temperature	0.015	< 0.0001	0.016	< 0.0001

### 3.6 Testing costs of extracted DNA from clinical swabs by *artus C. trachomatis* Plus RG PCR, qPCR and ddPCR

Overall costs and component parts can be found in Table 6. The commercial test a*rtus* was the most expensive at $25.04 per sample, and the least expensive was the Rotor-Gene at $9.51 per sample. Calculated costs include trained laboratory technician time for a Tanzanian Junior Laboratory Technician and therefore all testing runs can be prepared within a reasonably short period of time. A single ABI qPCR plate of up to 88 samples run with four technical replicates (plus standards) takes an experienced operator approximately 1.5 h to prepare and 1.75 h to run. The operator time is increased for ddPCR to 2 h preparation time for up to 94 single reaction samples and 2.5 h run time. Time savings can be found in the use of the Rotor-Gene which takes approximately 1 h to prepare and 1 h to run 63 single reaction samples. The latter is comparable to the time required to test 70 samples by *artus*.

**Table 6:** qPCR assay costs.

Costing category	Assay platform cost (US$)			
	*artus*	ABI	ddPCR	Rotor-Gene
DNA extraction (kit & consumables)	4.70	4.70	4.70	4.70
PCR reaction mix (inclusive of oligos)	17.18	1.70	2.84	1.10
Lab consumables	0.41	0.12	1.84	0.35
Personnel monthly salary multiplied by 1.2 for overheads and divided by 160 for hourly rate (based on a trained laboratory technician from Moshi, Tanzania)	3.37	5.06	6.74	3.37
Total	$25.04	$11.51	$15.64	$9.51

NOTE: Purchase equipment costs for an ABI 7900HT, Rotor-Gene Q 5plex (equivalent to the 3000 which is no longer available for purchase) and ddPCR working platform are $62,445, $38,142 and $115,154 respectively.

## 4 Discussion

We evaluated a qPCR assay that detects *Ct* plasmid and genomic targets, whilst assessing specimen sufficiency with the presence of human DNA. The assay has a linear analyte response for all three targets that is reproducible across a wide dynamic range (10^0^–10^6^ copies/test) of both plasmid and chromosomal targets. The limits of reproducible detection for both *Ct* targets are below 10 targets per test, which is comparable to other non-commercial PCR tests (Pickett et al., 2005, West et al., 2014). The absolute sensitivity of the *omcB* and pORF2 tests is similar, however, due to the relative abundance of the plasmid target in clinical specimens, the plasmid test detects a lower absolute number of chlamydial equivalents and therefore has a superior diagnostic performance.

The total assay variance within-centre was consistently < 1 PCR cycle. There was significant variation within-run (*omcB* mean: 24.7%, pORF2 mean: 19.5%), suggesting that, where precise quantitation is required, specimens should ideally be run in multiple wells. Where a qualitative diagnostic result is sufficient, running assays in a single well would only result in diagnostic failure due to assay variability at very low loads. The between-laboratory variance is higher, which could be attributed to instrument differences between the 7900HT and the Viia 7 (laser excitation versus halogen light source), however the assay was highly linear on both platforms and the total variance on either target was < 1.5 cycles.

External quality control exercises that included masked testing were used to evaluate these qPCR assays. During this exercise we successfully identified *Ct* infection in a specimen that carried a well-characterised plasmid deletion (Ripa and Nilsson, 2007) (Table 4). An endogenous control target confirms that the specimen comes from a human and has been stored and processed in a way that has not compromised the DNA quality therefore differentiating between infection negative tests and assays that have failed through PCR inhibition or absence of a testable DNA analyte.

Diagnostic performance compared to a commercially marketed *Ct* diagnostic kit (*artus C*. *trachomatis Plus RG*) was good, offering sensitivity and specificity > 90%. The median load of the false negative specimens was much lower than the load of the dual positive specimens, with the exception of one specimen detected by *artus* with a C_q_ of 23 cycles that was not detected by ddPCR or either qPCR assay. Agreement between assays was not perfect for any of the tested pairings between ddPCR, qPCR and *artus*.

Comparative *omcB* load analysis between the qPCR assay and ddPCR assay showed high concordance, with discordant results occurring at or below the limit of detection of the qPCR assay. At such low concentrations, sampling volume limitation impacts on the reproducibility of a positive result. Targets are sporadically detected in samples where the analyte concentration is below the limit of detection and the likelihood of a positive and negative result is limited by dilution/concentration and fits a Poisson distribution (Schachter et al., 2006).

Assuming the level of technical replication described in this paper and UK list prices from 2017, the qPCR assay costs roughly $11.51 per sample, inclusive of DNA extraction ($3.75), whereas testing using the commercial kit *artus* costs more than twice as much at $25.05 per sample. Reducing the number of technical replicates, reducing the volume of the assay to 5 μL, or omitting primer–probe sets for nondiagnostic targets (*omcB* or *RPP30*) could reduce the overall cost of the assay further, whilst the use of a larger DNA aliquot could offset potential loss of sensitivity. Use of the Rotor-Gene and ddPCR platform allows a larger sample volume to be assayed in a single reaction and is competitively costed against *artus* at, respectively, $9.51 and $15.64 per sample. Proprietary fluorophores may also be interchanged for non-proprietary equivalents to reduce cost or enable the assay to run on real-time thermocyclers from other manufacturers.

Along with other NAAT methods, qPCR is a useful research tool. In this study we utilize qPCR for three key purposes: (1) to determine the loss of material during extraction under differing conditions, (2) to determine the rate of degradation of DNA under different long-term storage conditions and (3) to analyse the concentration of diagnostic discrepant results. The BioChain extraction kit performed best in this study. For sample storage room temperature preservation rather than frozen, regardless of desiccation method, did not increase the rate of loss of detectable *Ct* DNA suggesting that control programs without access to a freezer may be able to store swabs at room temperature without loss of diagnostic performance. This has previously been described for *Ct* stored for long periods in transport media, and in short-term dry storage at room temperature (Gaydos et al., 2012, van Dommelen et al., 2013).

Together, these findings describe an optimal pipeline of sample handling and processing in a budget-conscious research setting. By demonstrating variability at each step of the pipeline, this study illustrates the flexible nature of qPCR that allows parameters to be modified according to user requirement (Peeling et al., 1992). The qPCR method described may offer an effective and affordable solution for quantitative estimates of *Ct* loads in trachoma studies.

## Contributions

Conceived the study: JH, RB, AR, TD, ChR, MJH.

Collected specimens: ARL, PAM, MJB.

Performed experiments: JH, RB, AR, BH, TD.

Analysed data: JH, RB, AR, TD, ChR, MJH.

Wrote manuscript: JH, RB, ChR, MJH.

Reviewed and approved manuscript: JH, RB, ARL, AR, BH, TD, PAM, MJB, ChR, MJH.
